# The Quality of Blood Donation Services and Its Association with Blood Donors’ Trust and Loyalty at Makkah Blood Donation Centers in Saudi Arabia: A Cross-Sectional Study

**DOI:** 10.3390/healthcare11152115

**Published:** 2023-07-25

**Authors:** Saeed M. Kabrah, Samer Abuzerr, Ruba Omar Almaghrabi, Raed Alserihi, Raed I. Felimban, Abdulrahman Mujalli, Akhmed Aslam, Bassem Refaat, Amr J. Halawani, Adel A. Alzhrani, Naif Samran AlMoteri, Fauziah Fawzi Abusaadh, Rasha A. Bulkhi

**Affiliations:** 1 Laboratory Medicine Department, Faculty of Applied Medical Sciences, Umm Al-Qura University, Makkah 24382, Saudi Arabia; ammujalli@uqu.edu.sa (A.M.); maaslam@uqu.edu.sa (A.A.); barefaat@uqu.edu.sa (B.R.); ajjhalawani@uqu.edu.sa (A.J.H.); 2 Department of Medical Sciences, University College of Science & Technology-Khan Younis, Gaza P.O. Box 8, Palestine; samer_516@hotmail.com; 3 Department of Social and Preventive Medicine, School of Public Health, University of Montreal, Montreal, QC H3C 3J7, Canada; 4 Department of Laboratory Medicine, Faculty of Applied Medical Sciences, Al-Baha University, Al-Baha 65431, Saudi Arabia; r.maghrabi@hotmail.com; 5 Department of Medical Laboratory Sciences, Faculty of Applied Medical Sciences, King Abdulaziz University, Jeddah 21589, Saudi Arabia; aaalserihi@kau.edu.sa (R.A.); faraed@kau.edu.sa (R.I.F.); 6 Center of Innovation in Personalized Medicine (CIPM), 3D Bioprinting Unit, King Abdulaziz University, Jeddah 21589, Saudi Arabia; 7 The Quality Department, Regional Lab and Central Blood Bank, Ministry of Health, Makkah 24321, Saudi Arabia; adalzhrani@moh.gov.sa; 8 Laboratory and Blood Bank Department, King Salman Abdul Aziz Medical City, Al Madinah 42319, Saudi Arabia; nsalmoteri@moh.gov.sa; 9 Haematology Department, Regional Lab and Central Blood Bank, Ministry of Health, Makkah 24321, Saudi Arabia; fwz1405@hotmail.com; 10 Public Health Department, Regional Lab and Central Blood Bank, Ministry of Health, Makkah 24321, Saudi Arabia; rbulkhi@moh.gov.sa

**Keywords:** blood donation services, loyalty, quality, Saudi Arabia, trust

## Abstract

The current cross-sectional study was conducted to determine the quality of blood donation services and its association with blood donors’ trust and loyalty at Makkah blood donation centers in Saudi Arabia. A total of 373 healthy blood donors aged ≥18 years who visited blood donation centers in Makkah, Saudi Arabia, between 1st and 28th February 2023 were recruited using a census sampling method. A pre-tested and validated Arabic language questionnaire was employed. The study survey included a checklist of sociodemographic variables (seven items), as well as seven-point Likert-scale questions on the quality of blood donation services (21 items), questions to assess the participant’s level of trust in blood donation centers (4 items), and questions to evaluate the level of loyalty to blood donations (4 items). SPSS (version 24) was used for data analysis. A total of 373 blood donors were included in this study. Of them, 240 (64.3%) were males and 133 (35.7%) were females. The vast majority of the study participants, 330 (88.5%), had a high educational level. The overall average agreement score for the quality of blood donation services was 71.7%. Furthermore, the overall average item agreement score for trust in blood donation centers and places was 83.0%, while the overall average item agreement score for loyalty to blood donation was 72.1%. Moreover, after adjustment for potential confounding factors, high levels of quality in blood donation services were associated with high levels of trust and loyalty among the blood donors (OR: 1.518, CI 95%: 0.321–0.864 and OR: 2.466, CI 95%: 0.285–0.763, respectively) (*p*-value < 0.05 for all). The overall quality of, trust in, and loyalty to blood donation services were 71.7%, 83.0%, and 72.1%, respectively. In addition, high levels of quality in blood donation services could improve blood donors’ trust and loyalty levels at Makkah blood donation centers in Saudi Arabia.

## 1. Introduction

The most priceless gift somebody can give to another person is their blood. If the blood is separated into components, giving blood can save one life or perhaps several [1]. In 2018, around 73% of reporting nations worldwide had a national blood donation policy. Overall, 66% of reporting nations, including 79% of high-income nations, 63% of middle-income nations, and 39% of low-income countries, have specific laws governing the quality and safety of blood transfusions [2]. The World Health Organization (WHO) advises that national blood supply networks should be well organized and integrated to manage all blood collection, testing, processing, storage, and distribution activities. The national blood donation system should be governed by a national blood policy and legal framework to encourage the consistent application of standards and uniformity in the quality and safety of blood products [3]. Significant efforts are being made to recruit and keep an acceptable number of regular, unpaid blood donors to provide a sufficient and safe blood supply [4]. Due to the small number of members of the appropriate population who wish to give regularly, hospitals have enduring concerns about their ability to meet the demand for blood from their patients [5]. Due to a rise in human life expectancy and the use of cutting-edge, aggressive surgical techniques that require significant amounts of blood and its components, the demand for blood and its ingredients is constantly increasing in many nations [6,7]. Therefore, blood banks are in charge of maintaining a balanced supply of and demand for blood. This can be achieved by offering blood donors and hospitals high-quality services [8].

The Saudi Arabian system for donating blood is a hospital-based blood banking system where blood banks are in charge of every aspect of the service, including finding donors; screening given blood for infectious agents; and preparing, storing, and dispensing components. The blood supply has substantially changed during the past three decades, moving from imported blood to locally recruited blood donors. A combination of involuntary donors (mostly patients’ families, friends, and co-workers) and an increasing number of voluntary, unpaid donors make up the blood supply. Through blood bank-organized donation campaigns, the latter group is quickly growing [9].

In addition, blood can only be transfused safely if it has been obtained from a donor who has been carefully screened, is healthy at the time of donation, has been tested for compatibility between their red blood cells and the antibodies in the patient’s plasma (as per national requirements), and has been screened for transfusion-transmissible infections. Additionally, a well-run blood transfusion service with high-quality systems and skilled healthcare professionals is required to guarantee the quality and safety of all blood and blood products throughout the process, from the choice of blood donors to administration to the patient [10,11].

Moreover, a product’s or service’s quality is crucial for gaining customers’ trust, loyalty, and satisfaction and for market share, profits, and growth [9]. In the service marketing literature, scholars and practitioners have paid close attention to service quality [12,13]. Numerous studies have been undertaken to gauge and evaluate service quality by establishing several service quality parameters [14,15]. Service quality dimensions are considered the criteria to assess quality in the service environment [16]. In the current study, participants’ opinions on the standard of blood donation services, their degree of trust in blood donation facilities, and their commitment to blood donations were solicited using a validated seven-point Likert-type scale. Service quality is seen as a model for customer loyalty, trust, and satisfaction [17].

Our understanding of Saudi Arabian blood donation services’ quality and loyalty and trust levels is limited. This is the first study to examine the relationship between the degree of service quality at blood donation centers in Saudi Arabia and blood donors’ loyalty and trust levels. The results of this study are essential for policymakers to ensure universal access to safe and sufficient blood and blood products. The findings will help to promote good transfusion practices and contribute to self-sufficiency with regard to safe blood and blood products based on voluntary, unpaid blood donation. This will ultimately help to achieve universal health coverage. Therefore, the current study was conducted to determine the quality of blood donation services and its association with blood donors’ trust and loyalty at Makkah blood donation centers in Saudi Arabia.

## 2. Materials and Methods

### 2.1. Study Design, Setting, and Period

A cross-sectional study was conducted using face-to-face, pre-tested, and validated Arabic language questionnaires among healthy blood donors who visited blood donation centers in Makkah, Saudi Arabia, between 1st and 28th February 2023.

### 2.2. Study Participants

In the current study, eligible healthy blood donors who visited blood donation centers in Makkah, Saudi Arabia, during the study period were recruited. The present study included all healthy blood donors of both genders aged ≥18 years who donated blood during the study period. Unhealthy participants, those aged below 18 years, and patients with blood-borne diseases, such as hepatitis B and C, HIV, etc., were excluded from the study in accordance with the blood donation criteria in Saudi Arabia.

### 2.3. Sample Size and Sampling Technique

In the current study, a total of 373 healthy blood donors of both genders aged ≥18 years who visited blood donation centers in Makkah, Saudi Arabia, during the study period were recruited using a census sampling method. The initial number of blood donors was 412. Among them, 39 were excluded from the study because they did not meet the inclusion criteria. The most common reasons for exclusion were distributed as follows: 21 donors were younger than 18 years of age, 11 were infected with hepatitis B, and 7 were infected with hepatitis C.

### 2.4. Data Collection

All participants were invited to answer a pre-tested and validated Arabic language questionnaire face-to-face. The study tool was developed and validated by Melián-Alzola and Martín-Santana in 2020 [18]. The study questionnaire included a checklist of sociodemographic variables (7 items), as well as seven-point Likert-scale questions on the quality of blood donation services (21 items), questions to assess the participant’s level of trust in blood donation centers (4 items), and questions to evaluate the level of loyalty to blood donations (4 items) [18]. Face and content validity were proved for the final draft of the Arabic questionnaire by seven experts in the field (researchers and health professionals) independently. Additionally, the relevancy rate of the questionnaire items was calculated using the content validity index as described in [19]. The final questionnaire was constructed by including all items that were relevant, with minor changes in the language. Subsequently, the survey was piloted among 20 participants, and the pilot results indicated an excellent overall Cronbach’s alpha of 0.85.

### 2.5. Pilot Study

Before the data collection process, a pilot study was undertaken among 20 participants to ensure the survey’s acceptance and consistency. After that, minor adjustments were made considering the pilot study’s findings.

### 2.6. Ethical Consideration

The study protocol was approved by the Umm Al-Qura University Biomedical Research Ethics Committee (no. HAPO-02-K-012-2023-01-1391). In addition, informed consent was obtained before participation from each participant. No monetary rewards were given for completing the questionnaire.

### 2.7. Statistical Analysis

Statistical Package for Social Science (SPSS, version 24) was utilized for analysis. Descriptive statistics were used to describe the characteristics of the study participants. The categorical variables were described using frequencies and percentages, and continuous variables were represented using mean values ± standard deviations (SDs). The chi-square test was used to determine the significant differences between categorical variables. A *p*-value < 0.05 was considered statistically significant.

## 3. Results

A total of 373 participants were recruited for this study. The study sample comprised 240 (64.3%) males and 133 (35.7%) females; 112 (30%) were between 18 and 25 years old. Most of the study participants (94.1%) were originally Saudi. A total of 219 (58.7%) participants were married, and 330 (88.5%) had a high educational level. Furthermore, 48.5% of the participants were government sector employees, and 43.2% had a monthly income of less than SAR 8699. In addition, for the variables age, nationality, marital status, employment status, and monthly payment, statistically significant associations were found by gender (*p*-values < 0.005 for all) (Table 1).

The overall average weight for the quality of blood donation services in Makkah was 5.4 ± 1.6. The highest average weight for the quality of blood donation services (6.0 ± 1.2) was for the item “the staff in the donation rooms (blood banks) is friendly and polite”. The lowest average weight for the quality of blood donation services (4.3 ± 2.1) was for the item “Donation centers or places (whether fixed or mobile) that provide parking for donors”. Additionally, the overall average item agreement score for the quality of blood donation services was 71.7% (Table 2).

The average weight for trust in blood donation centers and places was 5.6 ± 1.4. The highest average weight for trust in blood donation centers and places (5.7 ± 1.3) was for the item “I trust that the donation center or facilities always use blood appropriately”, whereas the items “I trust that the donation center or place is always working to ensure that patients have an adequate blood supply” and “I trust that a donation Centre or place always operates ethically” had an average weight of 5.6 ± 1.3. In addition, the item “I am confident that the donation center or facilities do not pressure donors to donate blood” had an average weight of 5.6 ± 1.5. Furthermore, the overall average item agreement score for trust in blood donation centers and places was 83.0% Table 3.

The overall average weight for loyalty to blood donation was 5.4 ± 1.7. The highest average weight for loyalty to blood donation (5.8 ± 1.5) was for the item “I encourage my relatives, friends, and co-workers to donate blood”. The lowest average weight for loyalty to blood donation (4.8 ± 2.1) was for the item “I will donate blood in the next four months”. Moreover, the overall average item agreement score for loyalty to blood donation was 72.1% (Table 4).

The crude and adjusted odds ratios and 95% confidence intervals for the quality of blood donation services and its relation to blood donors’ trust and loyalty were as follows. After adjustment for potential confounding factors, a high level of quality in blood donation services was associated with high levels of trust and loyalty among the blood donors (OR: 1.518, CI 95%: 0.321–0.864 and OR: 2.466, CI 95%: 0.285–0.763, respectively) (*p*-value < 0.05 for all) (Table 5).

## 4. Discussion

The current study aimed to determine the quality of blood donation services and its association with blood donors’ trust and loyalty at Makkah blood donation centers in Saudi Arabia.

The objective of evaluating service quality is to identify the factors and characteristics that account for experience quality and customer satisfaction in various contexts. There have been proposals suggesting a multidimensional structure for service quality [20]. Al-Zubaidi and Al-Asousi used a diagnostic investigation to identify critical areas based on the experiences of 354 donors and adapted the SERVQUAL scale to the donation setting. The categories included tangibles, dependability, responsiveness, assurance, and empathy [21]. Saha and Bhattacharya pursued similar goals, despite some of their traits being bad. They examined several characteristics, including tangibility, reliability, responsiveness, assurance, and empathy [22]. Jain et al. assessed the quality of customer service provided by blood donation banks and the relative weights donors assigned to the analyzed factors. The authors used the categories nonverbal communication, responsiveness, assurance, dependability, processes, and tangibles to form a seven-dimension structure [23].

Trust is the client’s confidence that the service provider will fulfill explicit and implied commitments to meet the donor’s expectations [24]. Trust (individual risk) is the belief held by the donor that their blood donation will have the desired result (social benefit) since the donation system will respect its obligations and that their actions will not adversely affect their health [25,26]. For these reasons, building trust is believed to be essential to reducing some of the risks and uncertainties that donors face [27]. Recently, Chen examined donation intention and behavior using the expanded theory of planned behavior and added confidence in blood donation organizations into their study model. Evidence shows that confidence in donation centers promotes positive opinions on giving [26]. These conclusions are supported by the current study’s findings. Loss of trust should be researched to further explain the low repeat rate among first-time contributors. Furthermore, a lack of trust may also be used to explain contributors’ generally dismal repeat rates.

Further findings from the present study were that 77.8% of participants talked to their family, friends, and coworkers about the benefits of blood donation; 70.7% said they would want to donate blood regularly (two or more times a year); and 57.6% said they would donate blood within the next four months. Increasing donor loyalty should be the system’s primary objective, as dedicated contributors are the ones that keep the contribution system in place [28]. To measure customer loyalty, several studies have employed factors like “intention to repeat” and “recommend the company to other people” [29,30]. Additionally, Boenigk and Helmig described the willingness of an individual to contribute more regularly and donate more money as an example of donor loyalty and suggested that donor loyalty included recommending that friends and relatives donate as well [31].

The major findings of the current study also showed that excellent quality in blood donation services might increase the loyalty and confidence of blood donors at Saudi Arabian blood donation centers. The literature supports the assertion that superior customer service comes before consumer trust. We may point to studies by Osman and Sentosa [24] and Eisingerich and Bell [32] as evidence. These studies support the beneficial effect of high service standards on clients’ faith in a company. Andaleeb and Basu showed how donation centers’ high standards help donors feel more confident. Therefore, having clean facilities, friendly staff, and a professional look, among other things, aid in preserving donors’ confidence [33]. The literature also supports the premise that great service comes before loyalty. Higher service quality boosts customer loyalty when using collaborative platforms according to Priporas et al. [34].

Furthermore, Parasuraman et al. demonstrated how service quality influences customer loyalty [20]. Additionally, Veerus et al. proposed that, if a donor has a negative first-time experience, the probability that they will donate again will drop [35]. Sargeant and Woodliffe [36] supported the idea that the caliber of the donor experience affects loyalty to the cause and the level of commitment.

Martn-Santana and Beerli-Palacio also provide empirical evidence of the positive effect that donor trust has on donors’ intentions to donate blood in the future. However, superior customer service and donor trust may favor donors’ loyalty to the donation center [28]. The association between trust and customer loyalty has been shown in many studies [37]. Sharma and Sharma noted that businesses must develop trust throughout their client relationship to guarantee repeat business [38]. Furthermore, Aldas-Manzano et al. concluded that trust is essential in dangerous situations like blood donation [39]. Sundermann used research to show how, in this context, trust influences donor loyalty [25]. More research is necessary to completely comprehend the relationships between blood donors’ levels of loyalty and trust and the caliber of blood donation services.

Finally, the quality of blood donation services should be frequently monitored using standard methods. In addition, national blood supply services should be well organized and integrated to manage all blood collection, testing, processing, storage, and distribution activities.

Globally, blood donation systems should be governed by national blood policies and legal frameworks to maintain the high quality of blood donation services and to promote trust and loyalty among blood donors.

The main limitation of this study is its cross-sectional methodology, which restricts the generalizability of our findings because a causal link cannot be established. In contrast, the study’s major strength is that it is the first to investigate the standard of blood donation services and its correlation with blood donors’ loyalty and confidence in Makkah, Saudi Arabia.

## 5. Conclusions

The overall quality of, trust in, and loyalty to blood donation services were 71.7%, 83.0%, and 72.1%, respectively. In addition, high levels of quality in blood donation services could improve blood donors’ trust and loyalty levels at blood donation centers in Saudi Arabia. Additional efforts are advised to develop a national blood system that can ensure prompt, universal access to safe and sufficient supplies of blood and blood products, as well as good transfusion practices that meet patient needs and progress towards self-sufficiency with regard to safe blood and blood products based on voluntary, unpaid blood donation.

## Figures and Tables

**Table 1:** Characteristics of the study participants by gender.

Variables	Total *n* (%) 373 (100.0)	Male (%) 240 (64.3)	Female (%) 133 (35.7)	*p*-Value
**Age (years)**				
18–25	112 (30)	33 (13.8)	79 (59.4)	0.001 *
26–35	81 (21.7)	47 (19.6)	34 (25.6)	
36–45	79 (21.2)	68 (28.3)	11 (8.3)	
>45	101 (27.1)	92 (38.3)	9 (6.8)	
**Nationality**				
Saudi	351 (94.1)	230 (95.8)	121 (91.0)	0.049 *
Non-Saudi	22 (5.9)	10 (4.2)	12 (9.0)	
**Marital status**				
Single	147 (39.4)	54 (22.5)	93 (69.9)	0.001 *
Married	219 (58.7)	183 (76.2)	36 (27.1)	
Divorced	7 (1.9)	3 (1.2)	4 (3.0)	
**Educational level**				
Low	4 (1.1)	3 (75.0)	1 (25.0)	0.062
Intermediate	39 (10.5)	18 (46.2)	21 (53.8)	
High	330 (88.5)	219 (66.4)	111 (33.6)	
**Employment status**				
Government sector employee	181 (48.5)	153 (63.8)	28 (21.1)	0.001 *
Private sector employee	48 (12.9)	37 (15.4)	11 (8.3)	
I do not work	144 (38.6)	50 (20.8)	94 (70.7)	
**Monthly income (SAR)**				
0 to 8699	161 (43.2)	63 (26.2)	98 (73.7)	0.001 *
8700 to 1199	48 (12.9)	38 (15.8)	10 (7.5)	
1200 to 15,299	71 (19)	57 (23.8)	14 (10.5)	
15,300 to 20,159	47 (12.6)	44 (18.3)	3 (2.3)	
20,160 or more	46 (12.3)	38 (15.8)	8 (6.0)	

* The difference was significant at the 0.05 level (two-tailed). Low education means illiterate or primary education; intermediate education means preparatory or secondary education; high education means a diploma or university education.

**Table 2:** Quality of blood donation services item scores.

Items	Strongly Disagree (%)	Disagree (%)	Somewhat Disagree (%)	Either Agree or Disagree (%)	Somewhat Agree (%)	Agree (%)	Strongly Agree (%)	Item Agreement Percent (%)
Donation centers and facilities provide appropriate advertisements and signboards to motivate blood donors **Average weight (mean ± SD): 4.9 ± 1.7**	3.8	8.0	7.8	17.2	22.3	11.8	29.2	63.3
Donation facilities provide privacy during the interview and donation **Average weight (mean ± SD): 5.3 ± 1.6**	3.5	3.5	6.2	18.0	17.2	17.2	34.6	69.0
The facilities for the donation are clean enough **Average weight (mean ± SD): 5.7 ± 1.4**	1.1	2.4	2.7	15.5	13.9	23.1	41.3	78.3
The donation facilities are intimate and comfortable **Average weight (mean ± SD): 5.4 ± 1.5**	1.1	2.7	8.8	17.2	14.7	19.3	36.2	70.2
Arrival at a donation center or place (stationary or mobile) is easy **Average weight (mean ± SD): 5.3 ± 1.6**	4.0	2.7	6.7	15.8	16.1	17.2	37.5	70.8
Donation centers or places (whether fixed or mobile) are available **Average weight (mean ± SD): 5.5 ± 1.5**	1.6	3.2	7.5	13.9	13.9	21.4	38.3	73.6
Donation centers or places (whether fixed or mobile) that provide parking for donors **Average weight (mean ± SD): 4.3 ± 2.1**	15.5	11.5	8.0	17.7	10.7	11.5	24.9	47.1
The working times (schedule) of the donation centers or places are appropriate **Average weight (mean ± SD): 5.0 ± 1.7**	4.3	6.4	8.3	18.2	16.4	16.6	29.8	62.8
The waiting time in the donation rooms before drawing blood is at least half an hour **Average weight (mean ± SD): 5.1 ± 1.6**	4.8	2.4	5.1	23.3	15.3	19.8	29.2	64.3
The duration of the blood donation process is appropriate **Average weight (mean ± SD): 5.7 ± 1.4**	1.9	1.9	2.7	12.9	15.3	22.5	42.9	80.7
The overall performance of the blood donation room staff is good **Average weight (mean ± SD): 5.9 ± 1.2**	0.5	1.1	1.1	13.1	15.0	21.2	48.0	84.2
Staff always explains donation requirements and procedures and recommends preventing possible adverse effects after donating blood **Average weight (mean ± SD): 5.6 ± 1.4**	1.1	1.9	5.9	15.3	14.2	16.6	45.0	75.8
The staff in the donation rooms (blood banks) is friendly and polite **Average weight (mean ± SD): 6.0 ± 1.2**	0.3	2.1	2.1	10.2	13.1	20.9	51.2	85.2
The staff in the donation rooms always looks after the donors’ health **Average weight (mean ± SD): 5.8 ± 1.3**	0.3	1.1	4.8	12.1	14.5	22.3	45.0	81.8
The staff in the donation rooms inspires confidence while donating blood **Average weight (mean ± SD): 5.7 ± 1.3**	0.3	2.4	3.2	16.1	13.9	19.6	44.5	78.0
The staff in the donation rooms answered my questions thoroughly **Average weight (mean ± SD): 5.8 ± 1.4**	1.1	1.9	3.5	12.1	15.5	19.3	46.6	81.4
At the end of a blood donation, the staff thanked me **Average weight (mean ± SD): 5.6 ± 1.5**	2.1	2.4	6.7	13.4	12.1	19.3	44.0	75.4
At the end of the blood donation process, a snack was provided **Average weight (mean ± SD): 5.3 ± 1.7**	5.1	5.1	4.3	15.3	15.3	16.9	38.1	70.3
I get a thank you note after every completed blood donation **Average weight (mean ± SD): 5.0 ± 1.9**	8.6	5.6	4.3	18.8	14.5	13.1	35.1	62.7
Donation information and analysis results sent are helpful **Average weight (mean ± SD): 5.3 ± 1.8**	6.7	4.8	3.5	16.9	12.3	15.8	39.9	68.0
It is easy to understand the information sent from the test results **Average weight (mean ± SD): 5.0 ± 1.9**	8.8	5.4	4.3	17.7	13.4	17.7	32.7	63.8
**Total item agreement score:**								**71.7**

**Table 3:** Trust in blood donation centers and places item scores.

Items	Strongly Disagree (%)	Disagree (%)	Somewhat Disagree (%)	Either Agree or Disagree (%)	Somewhat Agree (%)	Agree (%)	Strongly Agree (%)	Item Agreement Percent (%)
The donation center or place continually works to ensure patients have an adequate blood supply Average weight ± SD: 5.6 ± 1.3	0.8	1.1	6.2	8.8	29.8	17.2	36.2	83.2
I trust that a donation center or place always operates ethically Average weight ± SD: 5.6 ± 1.3	1.1	0.5	5.1	9.9	30.0	16.4	37.0	83.4
I trust that the donation center or facilities always use blood appropriately Average weight ± SD: 5.7 ± 1.3	1.3	0.5	5.1	9.9	27.3	13.4	42.4	83.1
I am confident that the donation center or facilities do not pressure donors to donate blood Average weight ± SD: 5.6 ± 1.5	2.9	2.1	5.1	7.5	27.1	12.9	42.4	82.4
**Total item agreement score:**								**83.0**

**Table 4:** Loyalty to blood donation item scores.

Items	Strongly Disagree (%)	Disagree (%)	Somewhat Disagree (%)	Either Agree or Disagree (%)	Somewhat Agree (%)	Agree (%)	Strongly Agree (%)	Item Agreement Percent (%)
I will donate blood in the next four months Average weight ± SD: 4.8 ± 2.1	12.3	6.2	7.2	16.6	12.3	9.1	36.2	57.6
I want to become a regular blood donor (two or more times a year) Average weight ± SD: 5.4 ± 1.8	5.9	3.2	8.6	11.5	11.0	13.1	46.6	70.7
I encourage my relatives, friends, and co-workers to donate blood Average weight ± SD: 5.8 ± 1.5	2.4	0.5	5.9	8.8	16.6	10.7	55.0	82.3
I discuss the positive aspects of blood donation among my relatives, friends, and co-workers Average weight ± SD: 5.7 ± 1.5	2.7	1.9	4.8	12.9	15.0	12.9	49.9	77.8
**Total item agreement score:**								**72.1**

**Table 5:** Crude and adjusted odds ratios with 95% confidence intervals for how well blood donors are treated and the association with their loyalty and trust.

Variables		Statistical Tests			
		Crude OR (95% CI)	*p*-Value ^a^	Adjusted OR (95% CI) ^b^	*p*-Value ^a^
**Trust in the blood donation center or place**					
Low (%)	9.8	Ref	-	-	0.001 *
High (%)	90.2	1.234 (0.6631–0.385)	0.162	1.518 (0.321–0.864)	
**Loyalty to blood donation services**					
Low (%)	23.1	Ref	-	-	0.003 *
High (%)	76.9	1.198 (0.409–1.217)	0.084	2.466 (0.285–0.763)	

^a^ Statistical testing using binary logistic regression. ^b^ Adjusted for age, nationality, marital status, employment status, and monthly income. Ref, reference. * The difference was significant at the 0.05 level (two-tailed).

## Data Availability

Not applicable.
